# Punicalagin Green Functionalized Cu/Cu_2_O/ZnO/CuO Nanocomposite for Potential Electrochemical Transducer and Catalyst

**DOI:** 10.1186/s11671-016-1581-8

**Published:** 2016-09-05

**Authors:** X. Fuku, K. Kaviyarasu, N. Matinise, M. Maaza

**Affiliations:** 1 UNESCO-UNISA Africa Chair in Nanosciences/Nanotechnology, College of Science, Engineering and Technology, University of South Africa, Muckleneuk ridge, PO Box 392, Pretoria, South Africa; 2 Nanosciences African Network (NANOAFNET), Materials Research Department, iThemba LABS-National Research Foundation of South Africa, Old Faure Road, PO Box 722, Somerset West, 7129 Western Cape South Africa

**Keywords:** Nanocomposite, Electrochemical methods, *Punica granatum* L, Nickel substrate and green synthesis

## Abstract

A novel ternary *Punica granatum* L-Cu/Cu_2_O/CuO/ZnO nanocomposite was successfully synthesised via green route. In this work, we demonstrate that the green synthesis of metal oxides is more viable and facile compare to other methods, i.e., physical and chemical routes while presenting a potential electrode for energy applications. The prepared nanocomposite was characterised by both microscopic and spectroscopic techniques. High-resolution scanning electron microscopy (HRSEM) and X-ray diffraction (XRD) techniques revealed different transitional phases with an average nanocrystallite size of 29–20 mm. It was observed that the nanocomposites changed from amorphous-slightly crystalline Cu/Cu_2_O to polycrystalline Cu/Cu_2_O/CuO/ZnO at different calcination temperatures (room temperature-RT- 600 °C). The Cu/Cu_2_O/ZnO/CuO metal oxides proved to be highly crystalline and showed irregularly distributed particles with different sizes. Meanwhile, Fourier transform infrared (FTIR) spectroscopy confirmed the purity while together with ultraviolet-visible (UV-Vis) spectroscopy proved the proposed mechanism of the synthesised nanocomposite. UV-Vis showed improved catalytic activity of the prepared metal oxides, evident by narrow band gap energy. The redox and electrochemical properties of the prepared nanocomposite were achieved by cyclic voltammetry (CV), electrochemical impedance (EIS) and galvanostatic charge-discharge (GCD). The maximum specific capacitance (*C*_s_) was calculated to be 241 F g^−1^ at 50 mV s^−1^ for Cu/Cu_2_O/CuO/ZnO nanoplatelets structured electrode. Moreover, all the CuO nanostructures reveal better power performance, excellent rate as well as long term cycling stability. Such a study will encourages a new design for a wide spectrum of materials for smart electronic device applications.

## Background

In recent times, the union between nanotechnology and biology has shaped a new ground of nanobiotechnology that integrates the use of biological bodies (i.e., bacteria, fungi, viruses, yeasts and plants) in a number of chemical and physical processes [1–4]. Green synthesis of nanomaterials using plant extract has emerged as a facile and viable route to traditional (chemical and physical) methods [1–3, 5]. Syntheses of nanoparticles (NPs) through nanobiotechnology processes have a significant potential to boost NPs production without the use of harsh, toxic, and expensive chemicals [2, 5, 6]. Studies have established that biomolecules identified from biological organisms can be used to control the nucleation and development of the inorganic nanostructures [3, 4, 7]. Metal oxide nanocomposites have shown great interest in many scientific fields [5–8]. Among others, copper and zinc oxide nanocomposites have attracted more attention due to their unique properties [6, 9–14]. In this view, novel *Punica granatum* L- binary (Cu/Cu_2_O) and ternary (Cu/Cu_2_O/CuO/ZnO) nanoscale materials in one dimension such as nanocubes, nanorods and nanoplatelets have been prepared via the green route. Zinc oxide (ZnO) is an important n-type semiconductor with a bandgap energy of 3.37 eV, and it also has interesting chemical, acoustic, optical and electrical properties [9, 10, 15–17] whereas the p-type cuprous (I) oxide (Cu_2_O—band gap of ~1.2 eV) and cupric (II) oxide (CuO—band gap of ~1.7 eV) have shown massive significance in the fields of gas sensing, optoelectronics, catalysis and solar cells and exhibit good photoconductive, photovoltaic and in many other fields [6, 10, 18, 19]. The production of copper and zinc nanocomposite with controllable sizes, shapes and surface properties is vital for exploring copper-zinc-based nano-oxides for different applications. There are numerous reports on chemically and physically prepared nanocrystalline copper-zinc based oxides [1, 10, 15, 20]. Deraz et al. reported the synthesis and characterisation of new copper-based nanocomposite using glycine-assisted combustion method [7] while in his paper (Habibi et al.) [21] fabricated and characterised ternary CuO-ZnO-Cu_2_O nanocomposites by Sol-Gel Route at different temperatures [22]. Meanwhile, Sasmal and other produced ternary Cu_2_O-Cu-CuO nanocatalyst with astonishing activities for 4-nitrophenol reduction in aqueous solution and synthesised catalytic Cu/Cu_2_O NPs prepared in aqueous medium [23, 24]. However, as far as we know, there are no green prepared binary/ternary copper-zinc oxide nanocomposites which have been reported. Therefore, the first-time green synthesised Cu/Cu_2_O and Cu/Cu_2_O/CuO/ZnO nanocomposite reveals a new route of synthesising the binary and ternary metal oxides relative to the chemical/physically modified routes. In this work, the nanocomposite will be used as a potential transducer for various applications, i.e., energy storage devices and catalysis. Further, the investigation will dwell much on the structural, morphological and electrochemical properties of the prepared Cu/Cu_2_O/CuO/ZnO system. Lastly, it will include the determination of a plausible mechanism of interaction between the biological entities and the nanocomposites.

## Methods

### Reagents and Materials

From pomegranate (*Punica granatum* L) fruit, peels were sourced and used by cleaning and drying them in the sun for few days (2–3 days). Metal precursors such as zinc acetate, copper acetate, 15 mL polyesterine graduated tubes and 0.22 μm hydrophilic filters (Whatman) were used in the synthesis and characterisation of the metal oxides while potassium bromide (KBr), potassium hydroxide (KOH) and silver/silver chloride (Ag/AgCl, 3 M) were used in FTIR and electrochemical measurements. Materials and reagents were purchased from Sigma-Aldrich. The cleanliness of the working electrode was maintained using alumina micropolish (1.0, 0.3, and 0.05 μm alumina slurries) and polishing pads supplied by Buehler, IL, USA.

#### Synthesis of *Punica granatum* L-Cu/Cu_2_O/CuO/ZnO Nanocomposite

Reduced Cu/Cu_2_O/CuO/ZnO nanostructures were prepared through green process. Briefly, equimolar amounts of zinc acetate and copper acetate (8 g - (0.4 mol L^−1^)) were added into a 250 mL round bottle containing yellow peel extract of *Punica granatum* L or punicalagin-polyphenol (at 80 °C)—which acted as both reducing and capping/chelating agent help form radicals which aid in the formation of nanoscaled materials. A dark brown precipitate of copper oxide (CuO)/zinc oxide (ZnO) nanoparticles (NPs), pH 5)) was formed. The precipitate was washed (with distilled water) and collected via a combination of sonication (10 min) and centrifugation (10 min, 1000 rpm). Seventy percent yield of the nanopowder was achieved and was dried in an oven at 65 °C, 1 h. After crashing the prepared Cu/Cu_2_O/CuO/ZnO nanocomposite into a fine powder using mortar and pestle, the nanomaterials were annealed at different temperature (100, 200, 300, 400, 500, 500 and 600 °C) after which they were characterised by different microscopic and spectroscopic techniques.

#### Preparation of Binary Ni/Cu/Cu_2_O and Ternary Ni/Cu/Cu_2_O/CuO/ZnO Electrodes

Electrochemical characterisation of binary Ni/Cu/Cu_2_O and ternary Ni/Cu/Cu_2_O/CuO/ZnO were performed according to earlier reports but with minor adjustments. Briefly Ni substrate was thoroughly cleaned by sonication in absolute ethanol and deionised water (5 min), respectively, and finally rinsed with distilled water. The cleaned substrate was dried in an oven (5 min) for modification. Finally, the binary Ni/Cu/Cu_2_O and ternary Ni/Cu/Cu_2_O/CuO/ZnO solutions were respectively drop-casted (60 μL) onto the Ni substrate and was then dried at 50 °C for 15 min and followed by gently washing with de-ionised water to remove any loosely bound material. The electrodes were labelled as Ni/Cu/Cu_2_O and Ni/Cu/Cu_2_O/CuO/ZnO. Potassium hydroxide solution (2 M, pH 5) was used as the electrolyte for electrochemical measurements.

### Instrumentation and Apparatus

#### Instrumentation

Morphological studies and particle distribution of Cu/Cu_2_O/CuO/ZnO nanocomposite was carried out using high-resolution transmission electron microscopy (HR-TEM—Philips Technai-FE 12 TEM instrument operated at an accelerating voltage of 120 kV) and high-resolution scanning electron microscopy (HRSEM). The Cu/Cu_2_O/CuO/ZnO nanocomposite constituents were enabled using EDS and X-ray diffraction (XRD) performed using a Rigaku D/MAX-PC 2500 X-ray diffractometer. Structural properties and mechanism of the prepared nano-oxides were determined by using Fourier transform infrared spectroscopy (FTIR—on a Perkin Elmer spectrometer (Spectrum 100)), and fluorescence and UV-Vis measurements were performed using the ocean view fibre optics LED. Finally, a conventional three-electrode cell system containing KOH as the electrolyte was used for all electrochemical measurements (ECO CHEMIE-PGSTAT 302N, Metrohm). For the electrochemical set-up, Ni substrate (0.5 × 1 cm) was used as the working electrode, the auxillary electrode was a Pt wire and the reference electrode was a Ag/AgCl electrode with 3 M NaCl solution.

## Results and Discussion

### Morphology and Elemental Studies of the Nanocomposite

**Fig. 1:**
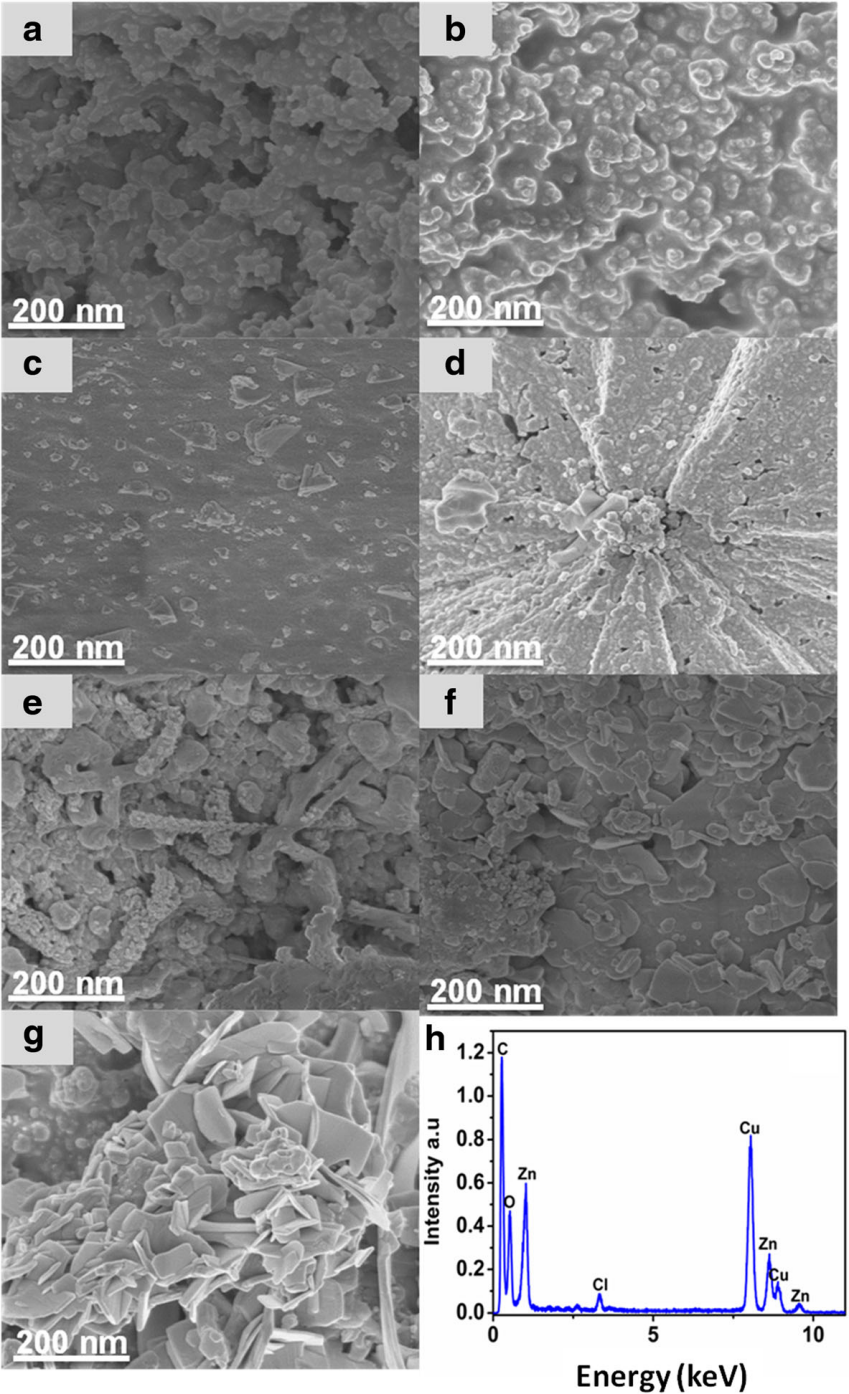
SEM images of as-prepared Cu/Cu_2_O/CuO/ZnO NPs (**a**) RT, (**b**) 100 0C, (**c**) 200 0C, (**d**) 300 0C, (**e**) 400 0C, (**f**) 500 0C, (**g**) 600 0C and (**h**) EDX spectra of the preapred Cu/Cu_2_O/CuO/ZnO NPs

Figure 1 shows different phase and structural morphology of the synthesised *Punica granatum* L-bimetallic oxides. Figure 1a–c reveals the morphologies of the p-type Cu/Cu_2_O nano-oxides ((at room temperature (Fig. 1a, 1b—100 °C and 1c—200 °C)), whereas Fig. 1d–g unveil different shapes of the prepared core shell Cu/Cu_2_O/ZnO/CuO ternary nano-oxides at different calcination temperatures (Fig. 1d—300 °C, 1e—400 °C, 1f—500 °C and 1g—600 °C). HRSEM images of Cu/Cu_2_O NPs revealed that the product which consists of spherical-like particles ((Fig. 1a, b) at RT and 100 °C)) changes to cube-like aggregate decorated with spherical nanoparticles (Fig. 1c at 200 °C). Meanwhile, the nano-oxides changed their phase and transition to more shell-like structures at higher temperatures (Fig. 1d—300 °C, 1e—400 °C, 1f—500 °C and 1g—600 °C). The SEM image of the prepared Cu/Cu_2_O/CuO/ZnO ternary metal oxides unveils the spherical-like structures decorated with a mixture of cubes and rods (Fig. 1d at 300 °C), changes to more rod-like particles festooned with spherical structures (Fig. 1e at 400 °C) and changes to platelets decorated with cube-like particles (Fig. 1e at 500 °C), and then, the ternary metal oxides changed to nanoplatelets with a fraction of spherical nanostructures (Fig. 1e at 600 °C). It is evident from the obtained results that a change in temperature affected the size, shape, morphology, phase, structure and formation of the nanocomposite. The structural change of Cu_2_O NPs is in good agreement with the results obtained by other researchers [5, 25, 26]. However, since it is the first time we produce ternary nano-oxides (Cu/Cu_2_O/CuO/ZnO) using green chemistry, there is no literature data to compare with except with sol-gel methods [21].

The synthesised cuprous oxide (Cu_2_O) NPs show an average particle size of 10 nm (Fig. 1a–c) while that of the prepared nanocomposites (Cu/Cu_2_O/CuO/ZnO) confirms an average crystalline size of 5–20 nm with the length and width of the nanorods (Fig. 1d, e) and nanoplateletes (Fig. 1f, g) ranging from 10 to 20 mm and 1 to 10 mm, respectively. Conclusively, the formation of the ZnO and CuO nanocomposite was fruitfully achieved, evident by different morphology and phase transitions. EDX was also employed to confirm the elemental composition of the prepared nanocomposite, Fig. 1h. Typical EDX plot revealed the main elements and confirmed that the synthetic route of (Cu/Cu_2_O/CuO/ZnO) was a success, evident by the presence of the main components at 1 and 8.6 keV (Zinc—Zn), 8.1 and 8.9 keV (Copper—Cu) and at 0.5 keV for oxygen (O). The observed energies are in agreement with other researchers [27–29].

### Structure and Crystallinity of the Prepared Nanocomposite

**Fig. 2:**
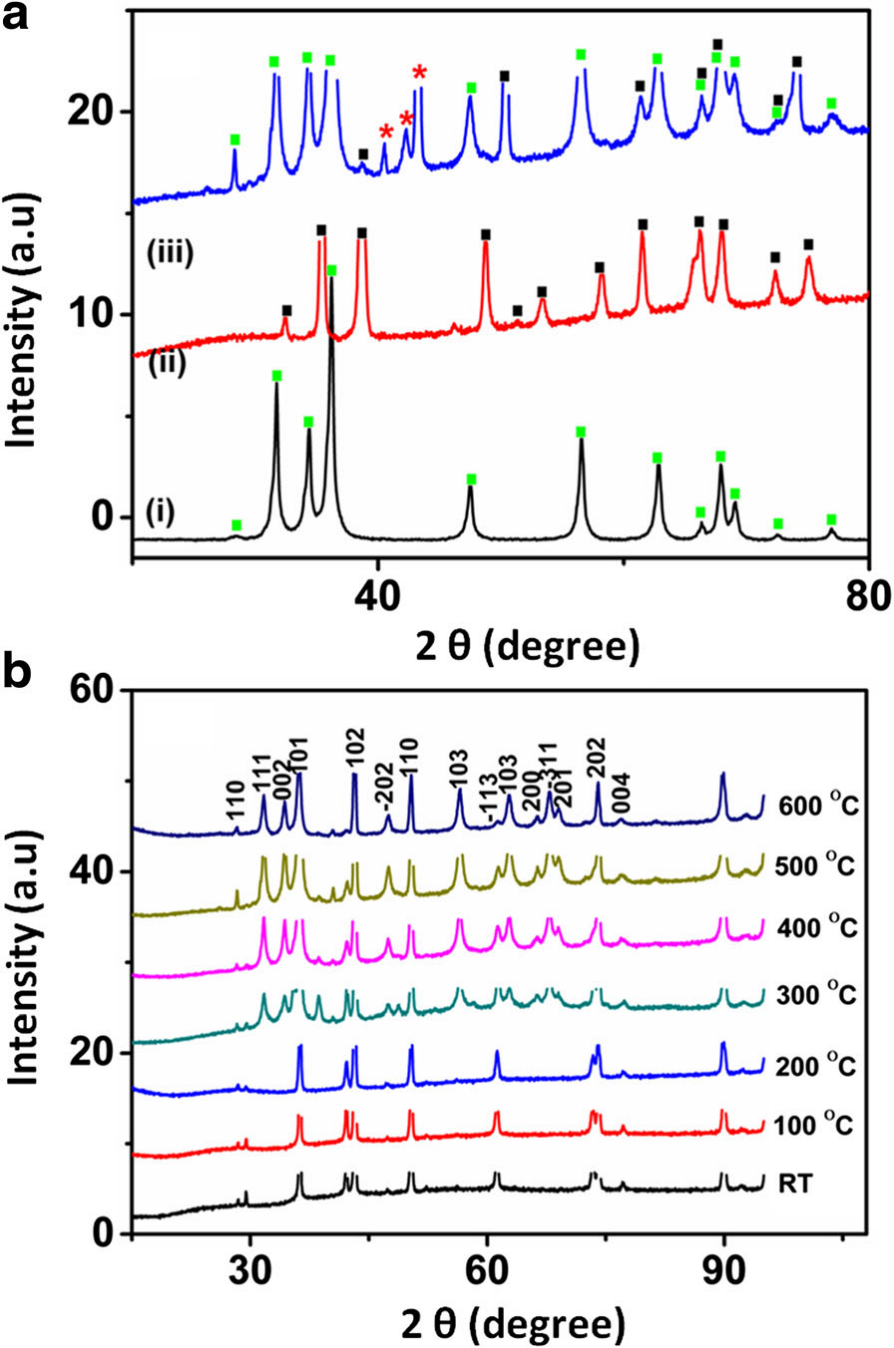
XRD spectra of **a** CuO (*i*), ZnO (*ii*), Cu/Cu_2_O (*iii*) and Cu/Cu_2_O/CuO/ZnO (*iv*) and **b** the as-prepared ternary nanocomposites at different calcination temperatures

**Fig. 3:**
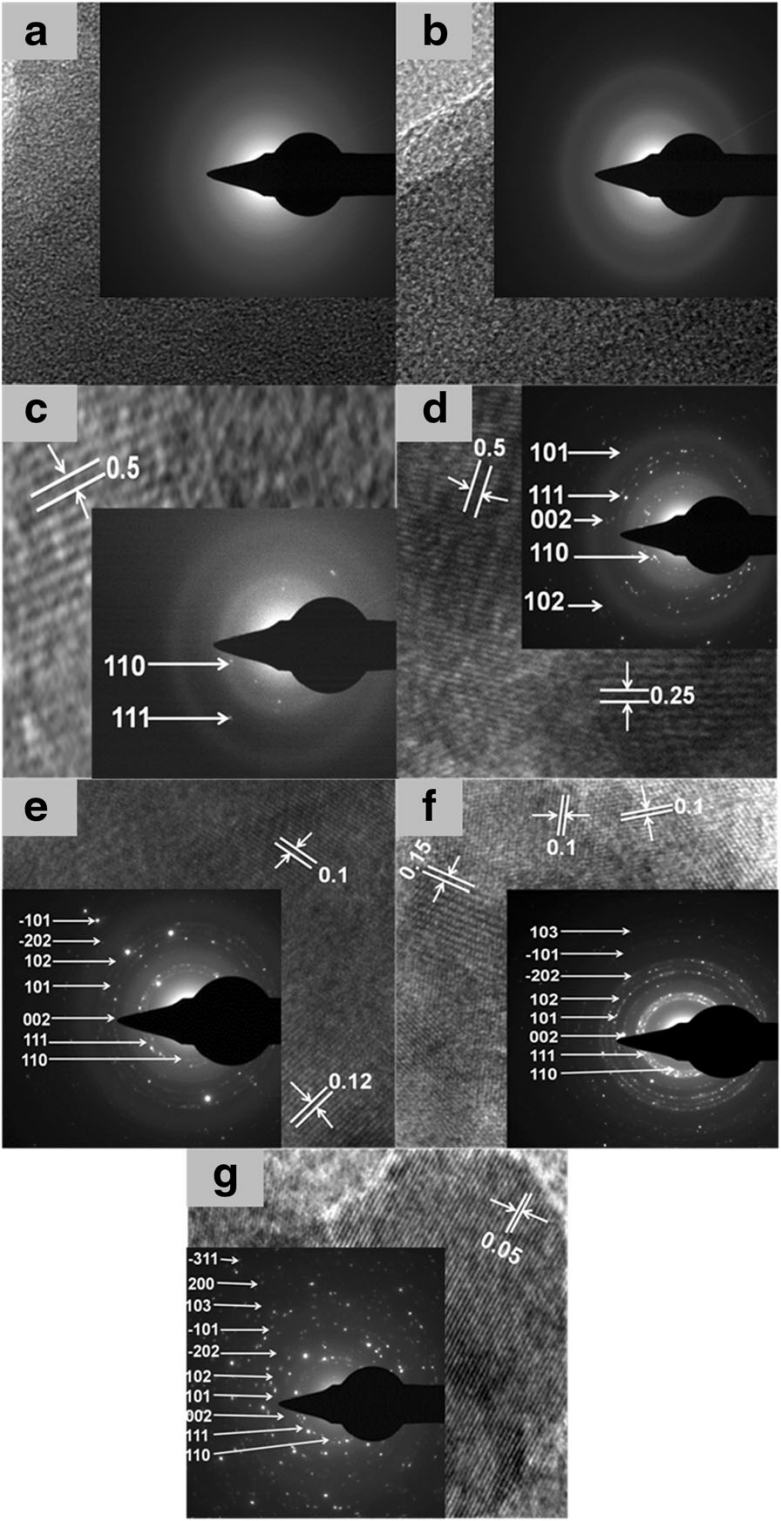
HRTEM images of as-prepared Cu/Cu_2_O/CuO/ZnO NPs at (**a**) RT, (**b**) 100 0C, (**c**) 200 0C, (**d**) 300 0C, (**e**) 400 0C, (**f**) 500 0C and (**g**) 600 0C

To study the crystalline nature of the prepared binary and ternary nanocomposite, XRD (Fig. 2) and HRTEM coupled with FFT (Fig. 3) were employed. Figure 2 shows XRD plots of the prepared nano-oxides at room temperature and after different calcination temperatures. From the observed results, the oxides display a pair of well-defined diffraction peaks implying their crystalline and nanoscale nature. For comparison, different metal oxide spectra (ZnO, CuO, Cu_2_O and nanocomposite) are displayed (Fig. 2a) and that of ternary metal oxide (nanocomposite only) at different temperatures are shown in Fig. 2b. Figure 2a(i) indicates the diffraction peaks of pure CuO NPs while those of pure n-type ZnO are shown in Fig. 2a(ii) accompanied by the p-type Cu/Cu_2_O (Fig. 2a(iii)) which finally forms the poly-crystalline ternary Cu/Cu_2_O/CuO/ZnO, Fig. 2a(iv). According to the XRD spectra, the prepared stable binary Cu/Cu_2_O were formed (at room temperature, 100 and 200 °C) proving that the biological route is more superior to the chemical and physical methods. The prepared sample clearly indicates the presence of two amorphous and crystalline phase indices: face-centred cubic (fcc) phase copper (Cu) and cubic phase cuprous oxides (Cu_2_O) at low temperatures (RT—200 °C), Fig. 2a(iii). The peak positions with 2θ values of 30.1°, 36.3°, 42.3°, 50.3°, 62.0°, 61° and 73.4° are indexed as (110), (111), (002), (101), (103), (004) and (200) planes which are in good agreement with those of powder Cu/Cu_2_O obtained from the International Centre of Diffraction Data card (JCPDS file no. 05-0667) confirming the formation of an amorphous fcc-single crystalline cubic phase Cu/Cu_2_O with a cuprite structure. In the interim, as we increase the temperature (300–500 °C), mixed phase Cu/Cu_2_O/CuO/ZnO nanocomposite becomes evident (Fig. 2b) with peaks corresponding to those of CuO, Cu_2_O, Cu and ZnO NPs, thus confirming the formation of the prepared ternary nanocomposite. The peaks which are denoted to both base-centred monoclinic CuO and hexagonal ZnO nano-oxides are discussed by Fuku et al. [30]. The XRD measurements revealed that the prepared sample consisted entirely of a mixture of CuO, Cu_2_O, Cu and ZnO phases. The XRD pattern of Fig. 2a(iii) consisted of amorphous tenorite Cu/Cu_2_O nanocomposite as a single-phase and well-crystalline Cu/CuO/Cu_2_O/ZnO particles as polycrystalline structure, Fig. 2a(iv). As viewed in Fig. 2a(iv), the sample contains different phases of copper crystallites that are Cu, Cu_2_O and CuO including ZnO. According to literature [29, 31], two copper oxides are able to form in air: CuO (tenorite) which is a stable oxide at room temperature, while the Cu_2_O (cuprite) is an intermediate copper phase. Therefore, the prepared sample was in harmony with other findings [1, 5, 23].

As the phase alteration of copper-zinc crystallites increases, the morphology of the NPs and nanocomposite has been tailored with subsequent decrease in the size (40–29 nm) of grains. The lattice parameters of the NPs were found to be hexagonal ZnO (*a* = 3.2, *b* = 5.2) and monoclinic cubic CuO/CuCu_2_O (*a* = 4.7, *b* = 3.4 and *c* = 5.1), and for the mixed phase, nanocomposites were found to be (*a* = 3.2, *b* = 5.2 and *c* = 5.1); thus, the results are in accord with the theoretical values. Using Scherrer’s equation [28], the calculated particle size and crystallite size (186–156 eV) of the ternary nanocomposite was found to be smaller than other prepared nano-oxides. Meanwhile, Fig. 3 confirms the diffraction patterns/micrographs of the preparated Cu-Cu_2_O and Cu/Cu_2_O/CuO/ZnO nanocomposites at different annealing temperatures (Fig. 3a–g: room temperature, 100, 200, 300, 400, 500 and 600 °C, respectively). High-resolution TEM images revealed multi-crystalline Cu/Cu_2_O/CuO/ZnO nanocomposite (Fig. 2c–g) relative to the amorphous-single crystalline Cu/Cu_2_O nanocomposite (Fig. 2a–b). The multiple-crystalline nature of the nanocomposite was observed as we increased the annealing temperatures (200–600 °C) and as the shape and the nanostructures were manipulated at a particular temperature. In addition, the micrographs also provided evidence of the lattice fringes and the d spacing of the synthesised nanomaterials. HRTEM images showed uni-directional lattice fringes (d spacing: 0.5 nm, Fig. 3c) and confirmed the mono-crystallinity of the binary nanocomposite (Cu/Cu_2_O). However, at higher temperatures, the ternary nano-oxide revealed multi-directional lattice fringes (Fig. 3d–g) with mixed d spacing (d: 0.05, 0.1, 0.15, 0.25 and 0.5) also illuminating the multi-crystalline nature of the prepared sample. From the obtained results, it is evident that when subjected to different calcination temperatures, the prepared nanocomposites expound different morphology, structural properties and crystallinity. Researchers such as Zhang et al. [32], Jiang et al. [10] and Chen [29, 31] found results slightly different to those obtained in this paper [23, 33–37], thus, confirming a significant and superior green synthetic route.

### Optical Band Gap Energy

**Fig. 4:**
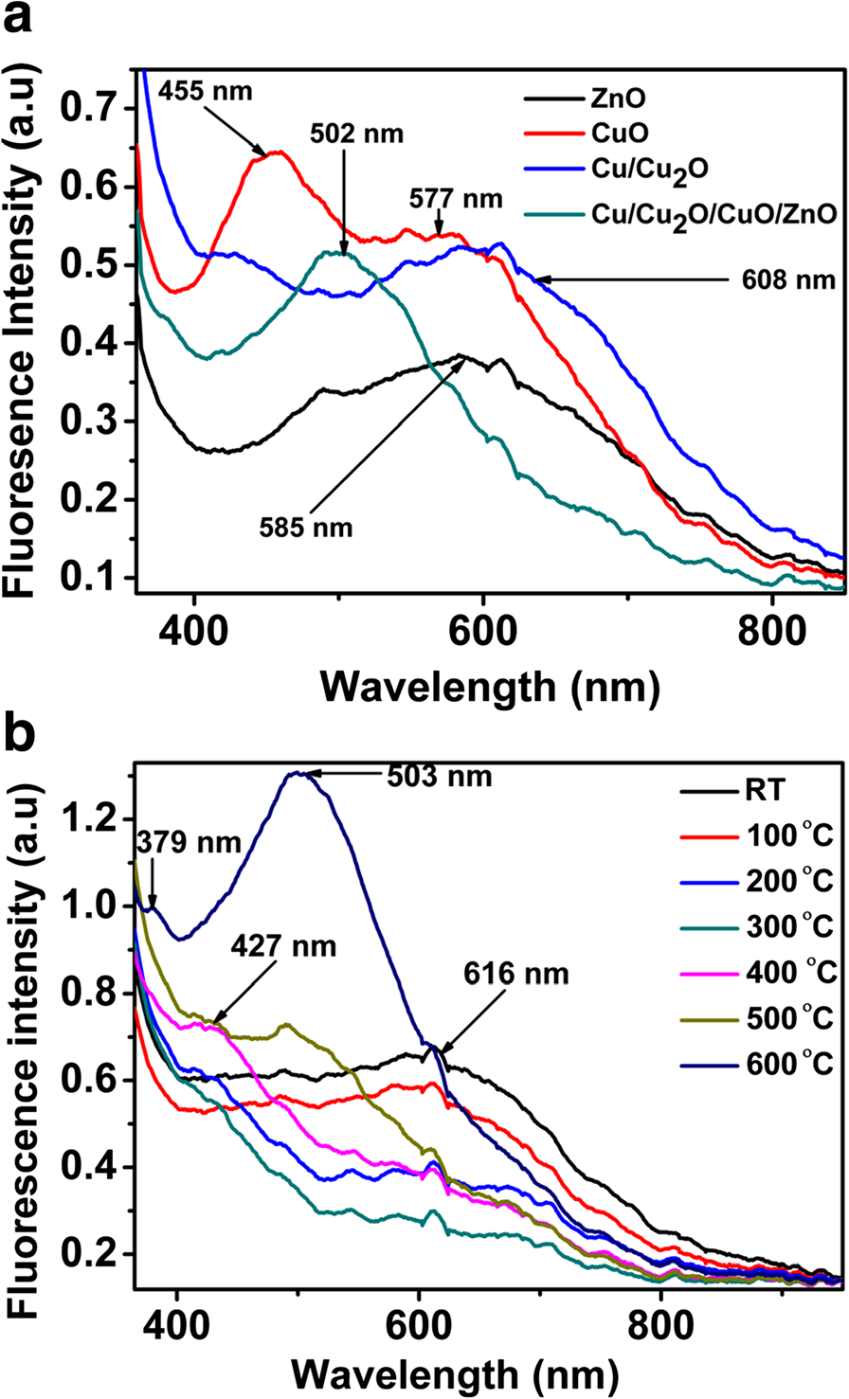
Fluorescence spectra of as-prepared (**a**) ZnO, CuO, Cu_2_O and Cu/Cu_2_O/CuO/ZnO and (**b**) Fluorescence spectra of as-prepared Cu/Cu_2_O/CuO/ZnO nanocomposite at different annealing temperatures

Fluorescence measurements were carried out to investigate the wavelength maxima (*λ*_max_) and the effect of temperature on the synthesised *Punica granatum* L-(CuO, ZnO, Cu/Cu2O and Cu/Cu2O/CuO/ZnO) NPs at 2 h intervals, Fig. 4a, b. Figure 4a reveals two CuO fluorescence peaks at *λ*_max_ of 455 and 608 nm. Further, the *λ*_max_ of ZnO was observed at 585 nm and that of Cu/Cu_2_O/CuO/ZnO was visible at 502 nm (Fig. 4a).

Figure 4b distinguishes the effect of temperature on prepared nanocomposite. The nanocomposite shows a broad emission peak at 608 nm at room temperature (RT) which decreases in intensity as we increase the temperature (RT—600 °C). The broad emission peak at 608 nm can be due to a combination of monoclinic CuO NPs and binary cuprite Cu/Cu_2_O nanocomposite. However, at 200 °C, a new emission peak develops at 427 nm (monoclinic cubic-centre CuO NPs), and it becomes more prominent with increasing temperature. Moreover, at 500 °C, the emission peak at 608 nm becomes blue shifted to 503-nm peak position. The emission peak at 503 nm can be assigned to the near band emission of the mixed phase Cu/Cu_2_O/CuO/ZnO nanocomposite. When subjected to high temperatures (600 °C), the peak at 503 becomes more intense and a new peak materialises at 379 nm which can be a blue shift of a peak at 427 nm. The increase in peak intensities can be attributed to the polycrystalline behaviour of the nanocomposite. Furthermore, the emission peaks are denoted to different phase transition present in the prepared sample. The spectra at high temperatures (500–600 °C) reveal three emission peaks (at 608, 503 and 379 nm) with three mix-phases, i.e., monoclinic CuO, fcc cuprite Cu_2_O and hexaganol tenorite ZnO NPs. The behaviour proves and confirms that at high temperature, there is indeed the formation of stable ternary nanocomposite (Cu/Cu_2_O/CuO/ZnO). The results are comparable with those obtained from XRD and TEM. In addition, the band gap energies of the prepared sample were calculated according to Max Planck’s equation as explained in our previous work [30]. The mission peak at 608 nm which shifted to 503 and merged into two emission peaks (427 and 379 nm) was used to calculate the band gap energy of the sample. The band gap energies of the NPs were found to range from 2.0, 2.4, 2.9 and 3.3 eV. Worth of note, the band gap energies of the prepared sample increased as we increase the calcination temperatures. From RT—200 °C, the smaller band energies due to electron shuttling required lesser energy to move from conduction band to the valence band and the reverse is true. The attained band gap energies were in proximity with those reported by other researchers [21, 24].

### Mechanism

**Fig. 5:**
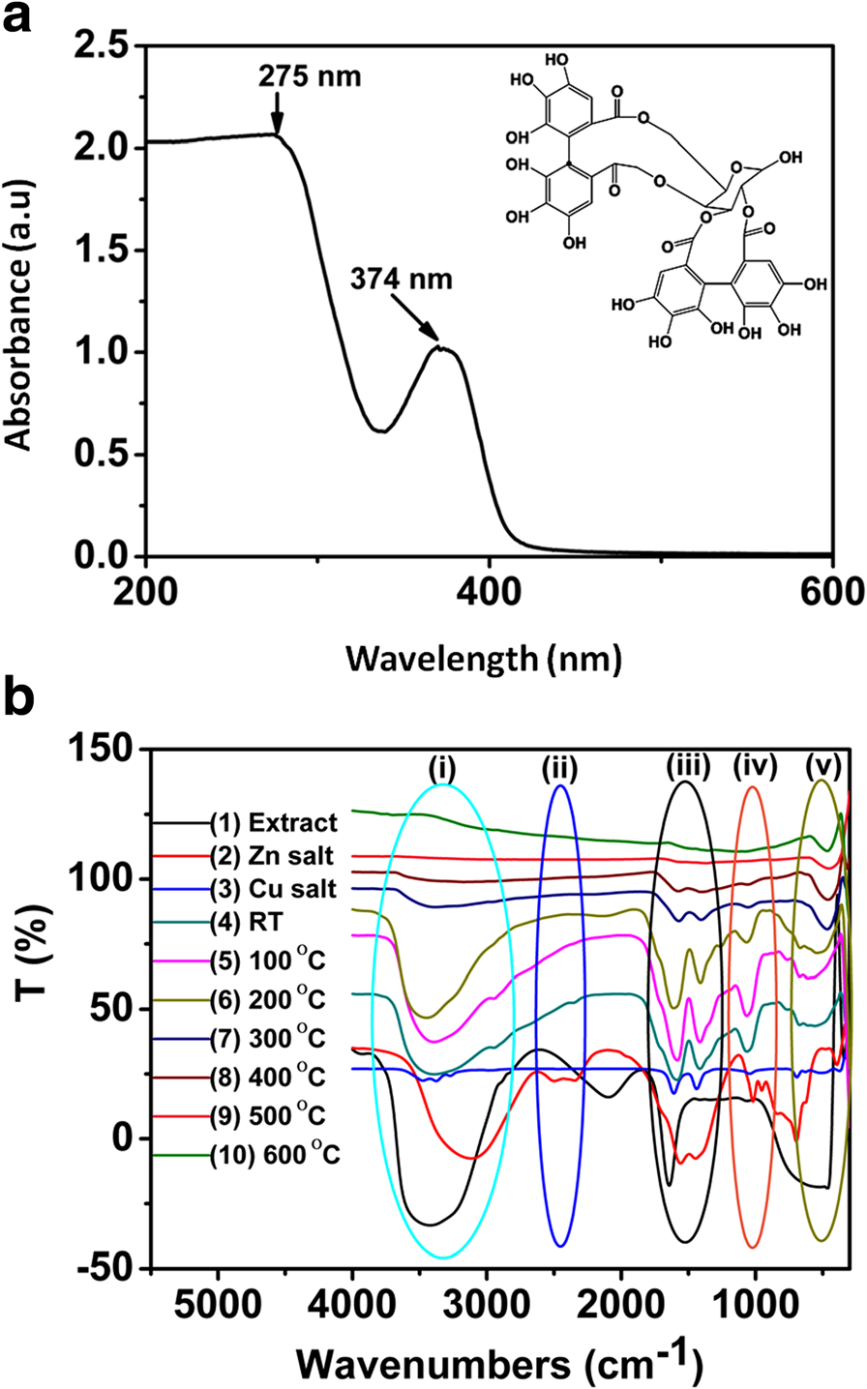
UV-Vis spectra of **a** Peel extract of *Punica granatum* L and **b** FTIR spectra of as-prepared Cu/Cu_2_O/CuO/ZnO NPs

**Scheme 1:**
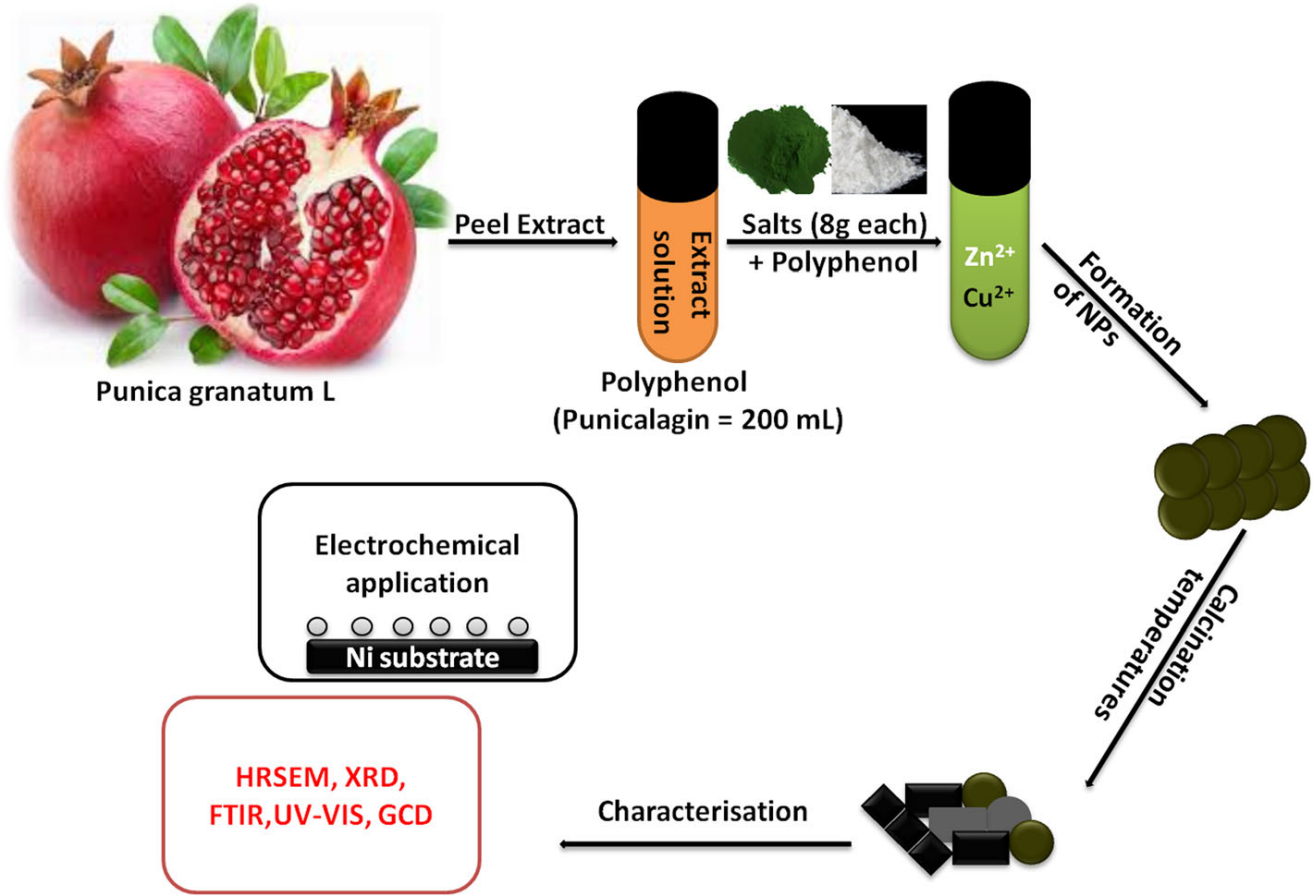
Schematic representation of the synthetic method/mechanism

FTIR and UV-Vis (Fig. 5b and Fig. 5a, respectively) were employed to ascertain the components and vibrational and absorption bands of the prepared samples. FTIR spectra (Fig. 5b) also provide us with the mechanism of interaction between the ligand and the complexes and bring evidences of coordination of Zn (II) and Cu (II) ions to the punicalagin molecule while UV-Vis (Fig. 5a) revealed the absorption wavelengths of the involved ligand (punicalagin) (Scheme 1).

At higher wavenumbers, the FTIR spectrum of the control (punicalagin), (Fig. 5b(1)) exhibits a broad band at 3500 cm^−1^ denoted to the strong intramolecular hydrogen bond (OH). An intense band was observed in the 1637 cm^−1^ neighbourhood and corresponded to the strong *ν*(C=O) and weak *ν*(C=C) stretching vibrations. Meanwhile, two slightly broad bands at 2000 and 600 cm^−1^ were noted and attributed to the δ(C=N), *v*(C=C=C) and *ν*(C–H) stretching vibrations. Compared to the control (Fig. 5b(1)), the IR spectra of the salts (Fig. 5b(2–3)) show slight shifts from high to low wavenumbers of the same vibrations. However, additional peaks were observed at lower regions of the precursors (Zn(OAc) and Cu(OAc)), 2501 cm^−1^ (not observed on the Cu(OAc) spectra) and 1007 cm^−1^. The observed peaks were due to the weak *ν*(O=C=O) and *ν*(C–O) stretching vibrations.

**Scheme 2:**
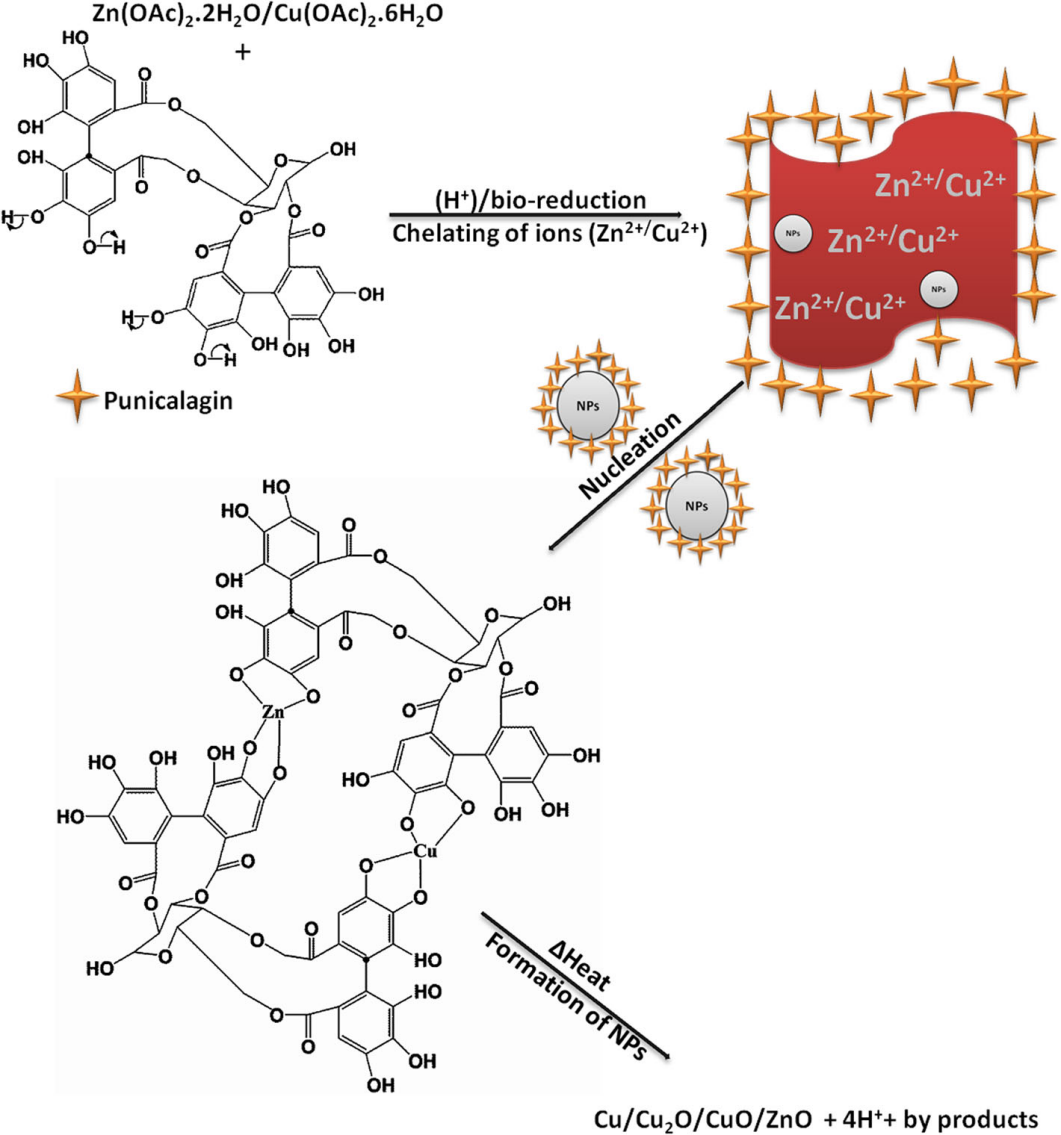
Possible mechanism of interaction between metal ions and punicalagin

After coordination of the precursors with the ligands, a possible mechanism of interaction was proposed (Scheme 2). Briefly, (Zn(OAc) and Cu(OAc)) dissociate into metal ion (Zn^2+^ and Cu^2+^) when in solution. Thus, phenolic compounds contained in the peel extract of *Punica granatum* L have hydroxyl and ketonic groups (in this case *punicalagin*) which are able to bind to metals and reduce the metal salt to a nanosize scale. After chelation of the ligands to the metal ions, coordination took place and a complex was formed (Zn/Cu-punicalagin). Furthermore, to substantiate this coordination and chelation, a comparison study of the FTIR spectra between, i.e., prepared nanomaterials, metal precursors and the extract was carried out, Fig. 5b. Compared to both the metal precursor and the control, the nanocomposite (Fig. 5b(4–5) (i–v)) shows a shift and a decrease in wavenumbers at 3450 cm^–1^ (O–H), 1600 cm^–1^ (C=O) and 600 cm^–1^, thus confirming the chelation and coordination of ligands to the metal ions. However, as we vary the annealing temperature (Fig. 5b(6–10)) the deformation of the (OH), *ν*(C=O) and *ν*(C–O) vibrational stretch was evident, suggesting deprotonation and formation of the pure Cu/Cu_2_O/CuO/ZnO at 458 cm^−1^ region, (Fig. 5b(8–10)). The FTIR spectra indicated an involvement of the hydroxyl group in the coordination while the metal ions displaced the H^+^ and form a complex.

### Electrochemical Properties

**Fig. 6:**
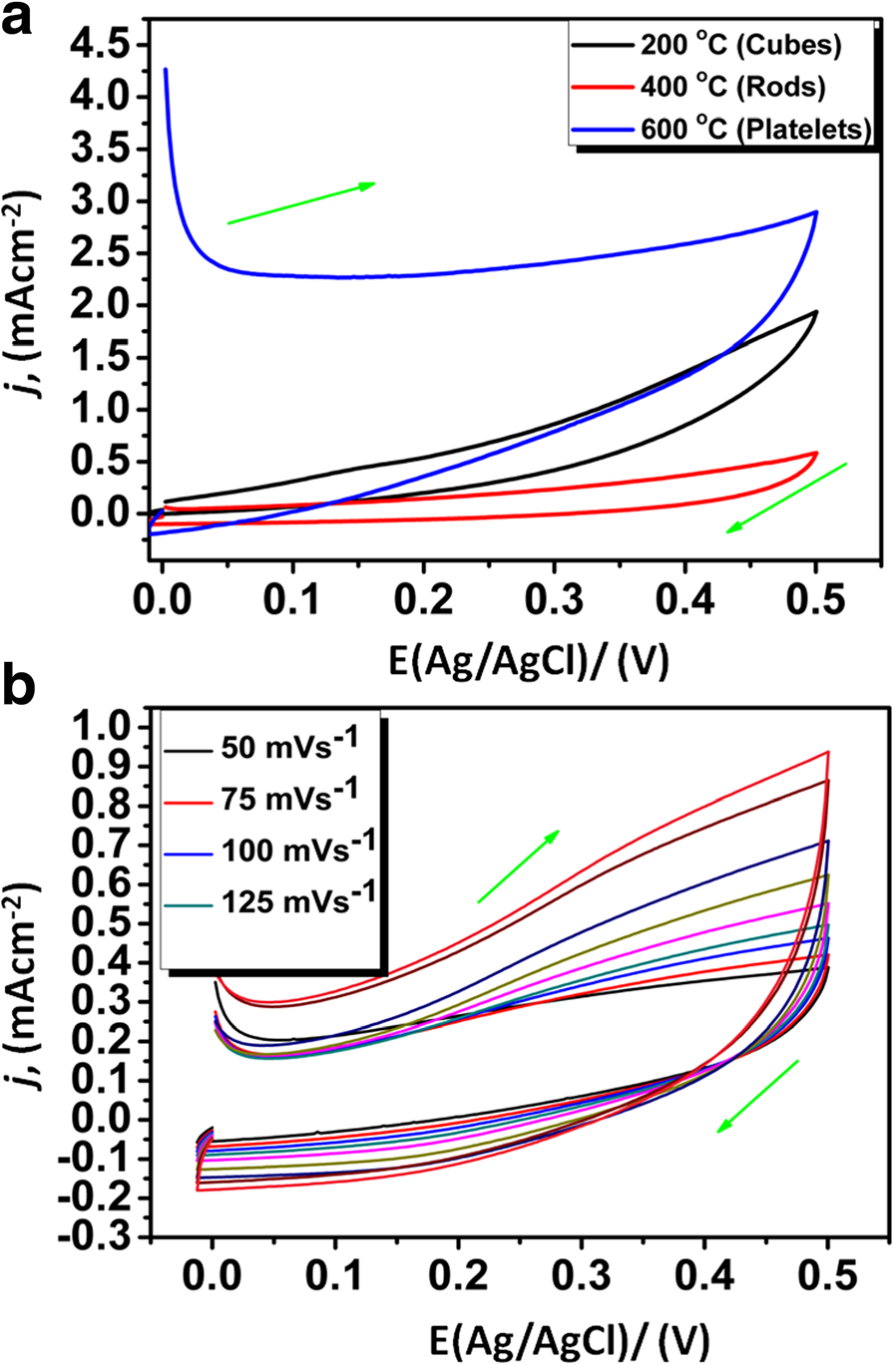
Voltammograms of **a** different structures of Ni/Cu/Cu_2_O/CuO/ZnO and **b** Ni/Cu/Cu_2_O/CuO/ZnO electrode in 2 M KOH at different scan rates (50, 75, 100, 125, 150, 175, 200 and 225 mV s^−1^)

**Fig. 7:**
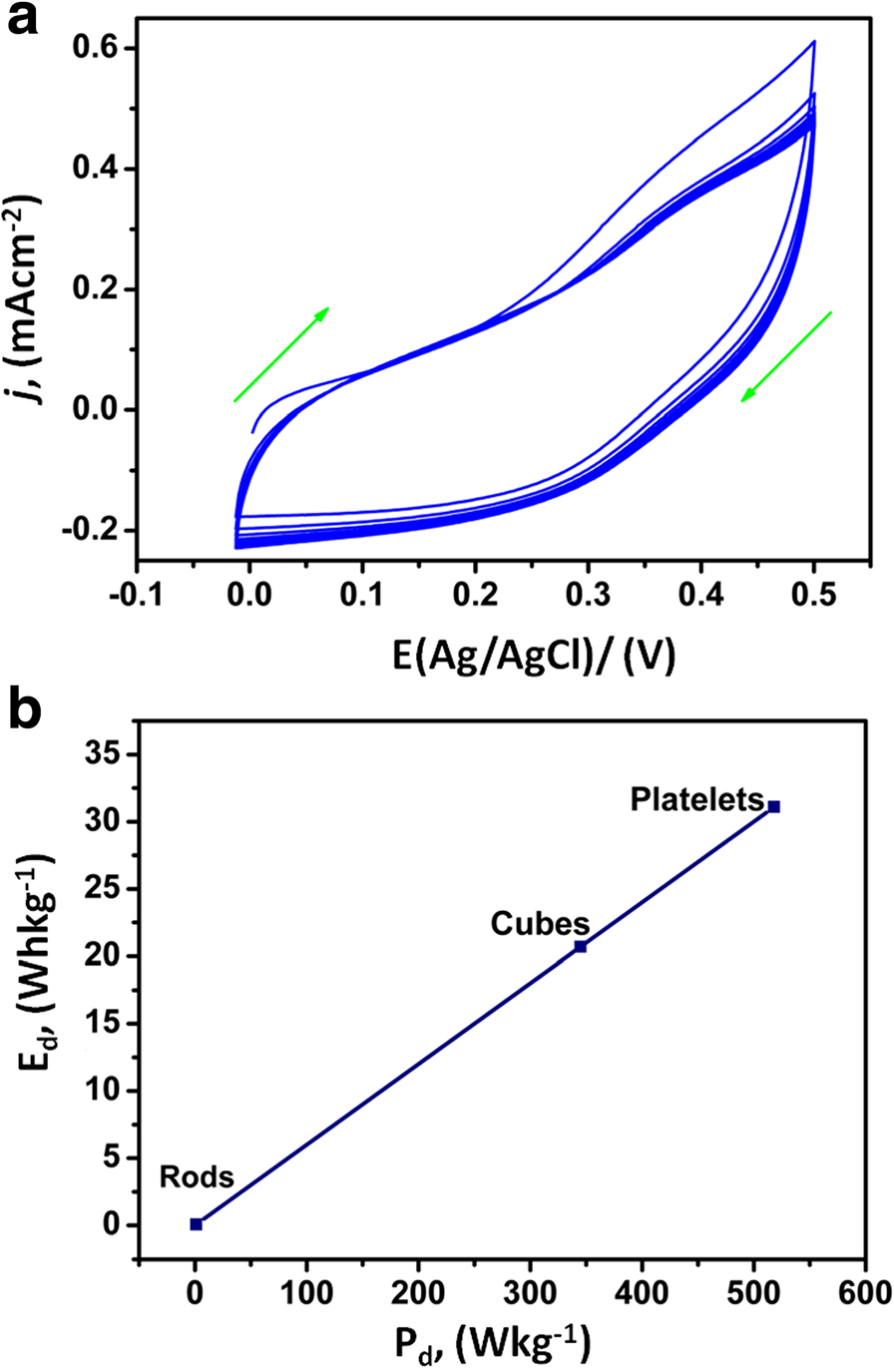
Voltammogramms of (**a**) Cubes (200 0C), Rods (400 0C) and Platelets (600 0C) and (**b**) Ni/Cu/Cu_2_O/CuO/ZnO electrode ate different scan rates

**Fig. 8:**
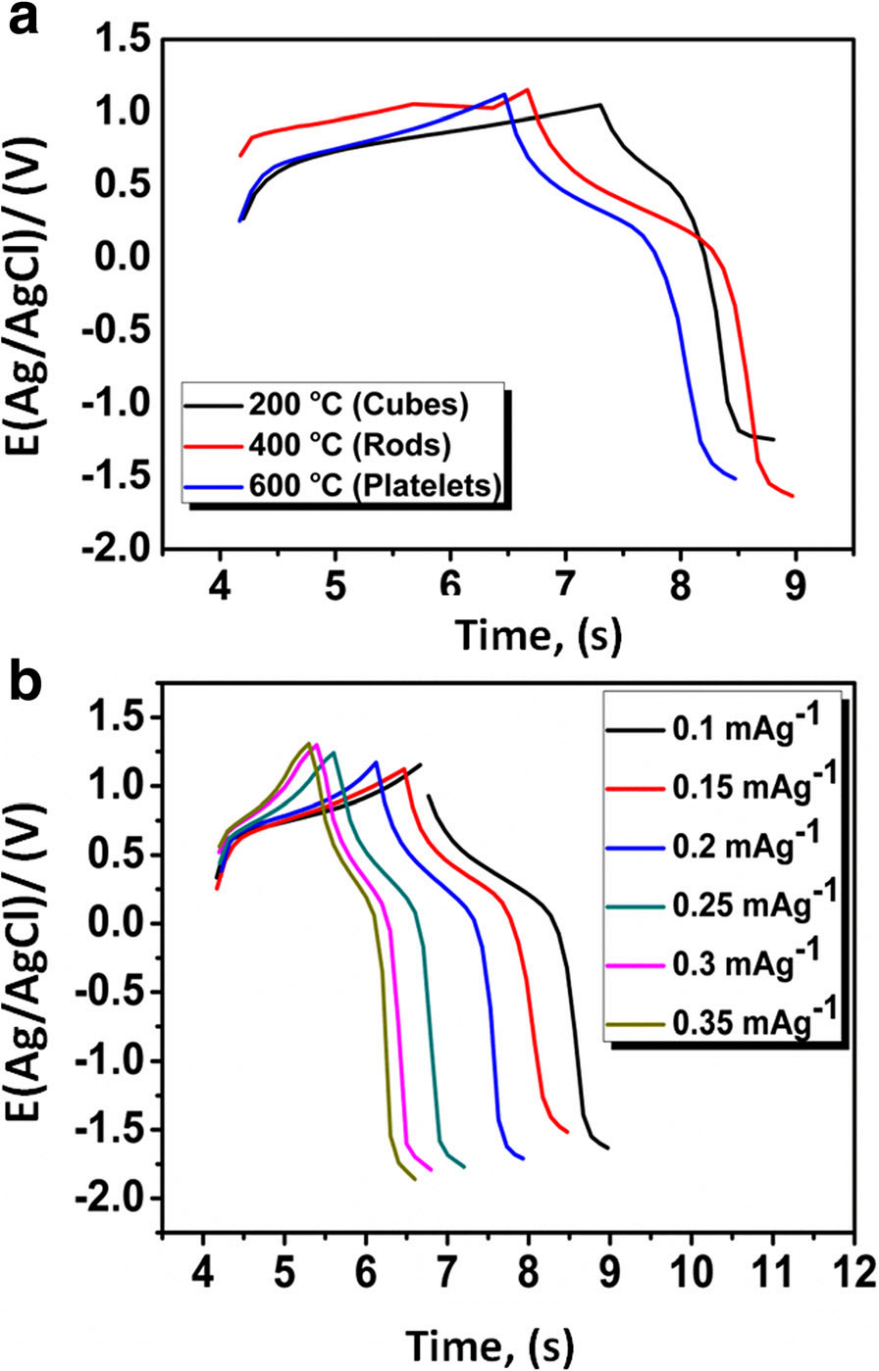
Charge-discharge curves of Ni/Cu/Cu_2_O/CuO/ZnO electrode in 2 M KOH at **a** constant current density (0.1 mAcm^−2^) and **b** at various current densities (0.1–0.35 mAcm^−2^)

**Fig. 9:**
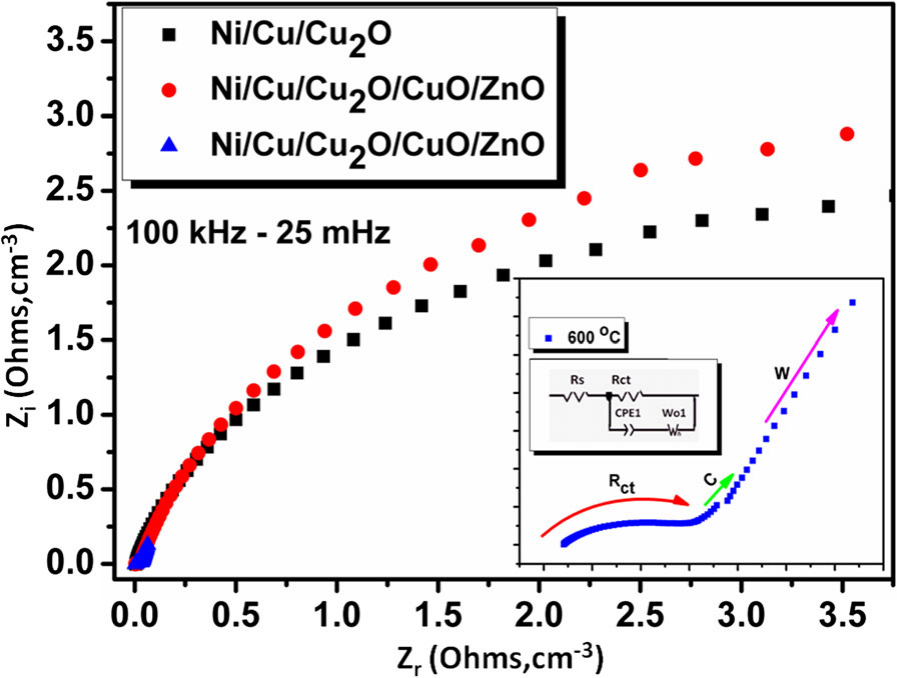
Nyquist plots of Ni/Cu/Cu_2_O/CuO/ZnO electrode in 2 M KOH at 50 mV

To ascertain the pseudo-capacitive nature of the electrochemically active binary and ternary nanocomposites (Cu/Cu_2_O and Cu/Cu_2_O/CuO/ZnO), different electrochemical methods such as cyclic voltammetry (CV), electrochemical impedance (EIS) and galvanostatic charge-discharge (GCD) were employed, Figs. 6, 7, 8 and 9. Figure 6a displays CV curves of the synthesized cubic-like, rod-like and platelet-like structure of the nano-oxides at a potential range of 0 to 0.5 V (vs. Ag/AgCl) using a scan rate of 50 mV s^−1^. For each curve, a broad redox background is evident signifying that the electrochemical mechanism is administered by pseudo-capacitive behaviour [38]. The *C*_s_ of cube-like, rod-like and platelet-like structure was estimated according to equation 1:

1 $$ {C}_{\mathrm{S}}=\frac{I_{\max }}{W\times \left(\mathrm{d}\mathrm{V}/\mathrm{d}\mathrm{t}\right)\left({V}_1-{V}_2\right)} $$

where *C*_s_ is the specific capacitance, dV/dt is the potential scan rate (mV s^−1^), *W* is the deposited weight, *V1* and *V2* are the starting and ending points of potential window and *I*_m_ is the current response (mA) of the nanomaterials on electrode for unit area (0.5 × 1 cm^2^). The calculated values of *C*_s_ at the electrode interface were found to be 241 F g^−1^ (platelets), 161 F g^−1^ (cubes) and 47 F g^−1^ (rods) and were comparable to other hydride electrodes [38] but superior to other reported values [38–43]. The nanoplatelet substrate carried the maximum *C*_s_, which may be due to its larger specific surface area and mixed hexagonal-monoclinic cubic structural geometry. The results substantiate that the electrode with the platelet-like structures was an effective transducer and can be used for further electrochemical analyses.

Figure 6b reveals the scan rate dependence studies of the ternary platelet-like nanostructures at the nickel-substrate interface (Ni/Cu/Cu_2_O/CuO/ZnO). The improvement in the reduction current as a function of scan rate suggests an effective use of the nanoplatelet modified electrode by the electrolyte, owing to better ionic diffusion. The enlarged area of the CV curve for nanoplatelets structured substrate corresponds to superior capacitance.

To further understand the stability and reproducibility of the transducer (Ni/Cu/Cu_2_O/CuO/ZnO), CV was employed, Fig. 7a. The stability and reproducibility of the transducer was done by keeping the electrode for 48 h in the dark after its redox properties were evaluated by CV. The electrode was cycled 40 times at a scan rate 50 mV s^−1^ with identical profiles. The multiple scans from the second scan reveals an increase in the anodic peak current and a decrease in cathodic peak current in which both the peak currents remind constant after the fifth cycle. It can be clearly stated that the formed electroactive species at the electrode interface remains adhered to the Ni substrate and the repeatability of the voltammograms also showed the durability of Cu/Cu_2_O/CuO/ZnO nanopowder at the electrode interface. Statistical analyses were performed based on the stability and reproducibility of the transducer. From the calculated standard deviation and mean values (not shown), the transducer only degraded by 3 %.

Figure 7b represents the two main parameters of power applications in electrochemical supercapacitors, i.e., energy density (*E*_d_) and power density (*P*_d_). The two applications including *C*_s_ are the main requisite of ultimate electrochemical supercapacitor at high charging-discharging rates. The *E*_d_ and *P*_d_ values of Ni/Cu/Cu_2_O/CuO/ZnO transducer were obtained from the CV measurements at 50 mV s^−1^ (Fig. 7b). The Ragone plot (*E*_d_ vs. *P*_d_) of Ni/Cu/Cu_2_O/CuO/ZnO_(nano rods)_, Ni/Cu/Cu_2_O_(nano cubes)_ and Ni/Cu/Cu_2_O/CuO/ZnO_(nano platelets)_ electrodes (Fig. 2b) was plotted and compared with the standard Ragone plot [39, 44, 45].

Compared to nanocubes and nanorods, nanoplatelet-structured electrode exhibits higher *E*_d_ (32 Whkg^−1^) with an increased *P*_d_ (550 Wkg^−1^) at 50 mV s^−1^ (Fig. 2b). The results suggest a supercapacitive behaviour of the nanoplatelets which are within limits of the standard Ragone plot [45, 46]. The results advocate employment of nanoplatelets structured electrode in the fabrication of supercapacitor devices. Meanwhile, the GCD was employed to confirm the capacitive profiles of the cube-like, rod-like and platelet-like morphological structures at constant current density of 0.1 mAg^−1^, (Fig. 8a). The charge-discharge (CD) curves (Fig. 7a) resulted in a nearly symmetrical behaviour with a slight curvature. The distortion/shape of the CD curves indicates the pseudo-capacitance nature of the prepared nanostructures and was utilised to assess the *C*_s_ of individual nanostructures from equation 2:

2 $$ {C}_{\mathrm{S}}=\frac{I\times t}{\varDelta V\times m} $$

where *t* is the discharge time, *m* is the mass of the active material on the electrode and ∆*V* is the potential range/window.

The *C*_s_ value of the cube-like Cu/Cu_2_O (at 200 °C) was calculated to be 106 F g^−1^, the rod-like Cu/Cu_2_O/CuO/ZnO (at 400 °C) has the *C*_s_ of 157 F g^−1^ and that of platelet-like Cu/Cu_2_O/CuO/ZnO (at 600 °C) was found to be 233 F g^−1^ at the same discharge current density. The results of the calculated *C*_s_ are similar to those calculated from CV.

Figure 8b shows the GCD profiles at various current densities (0.1–0.35 mAcm^−2^) exhibiting a reasonably symmetric curves. Potential (*E*) plateau in discharge curves was apparent at 0.3 V, which was in unison with the CV curves of Fig. 8a, b. As expected, the *C*_s_ of the nanostructured electrode decreases linearly with increasing current densities, which is the typical behaviour of electrochemical supercapacitors. The platelet-like structures showed a vital increment in the charge storage using the same electrochemical transducer (Ni/Cu/Cu_2_O/CuO/ZnO), Fig. 8b. The resultant *C*_s_ was attributed to the unique structure of the metal oxide with irregular distributions of Cu, O and Zn elements, increased number of oxidation states and ionic penetration in the electrode surface. Further, EI spectroscopy was employed to reveal the kinetics of Cu/Cu_2_O/CuO/ZnO at the electrode surface (Fig. 9). The nyquist plot which was recorded from 100–25 mHz revealed the electrical properties of Ni/Cu/Cu_2_O/CuO/ZnO (Fig. 8). The equivalent circuit (insert—Fig. 9) was used to evaluate and simulate the specific parameters (Rs: solution resistance, *W*: Warburg diffusion impedance, *R*_ct_: charge-transfer resistance and *C*: Faradaic pseudo-capacitance) as presented by the behaviour of our transducer. Remarkably, the nyquist plots reveal characteristic circular curvature of Ni/Cu/Cu_2_O (at 200 °C) and Ni/Cu/Cu_2_O/CuO/ZnO (at 400 and 600 °C) at a high-frequency region, signifying that the *R*_ct_ is prominent during charge-discharge process. Furthermore, the linearity or inclined line at the lower frequency regime denotes a capacitive behaviour of the electrodes whereas at high frequency, a diffusionally controlled system is apparent.

Through simulation, the charge-transfer resistance (*R*_ct_) values at the Ni/Cu/Cu_2_O, Ni/Cu/Cu_2_O/CuO/ZnO electrodes were found to be 256, 342 and 184 Ω, respectively. Comparatively, the Ni/Cu/Cu_2_O/CuO/ZnO (at 600 °C) proves to be favourable than other transducers, due to their lower *R*_ct_ values. The small *R*_ct_ proposes a faster electron shuttling, hence high conductivity at the electrode interface. The capacitive behaviour of the prepared transducer was determined by equation 3:

3 $$ \begin{array}{l}{Z}^{\hbox{'}\hbox{'}}={\left(2\pi fC\right)}^{-1}\\ {}{C}_{\mathrm{sp}}=\frac{-2}{2\left(\pi mf/{Z}^{\hbox{'}\hbox{'}}/\right)}\end{array} $$

where |*Z*′′| is the imaginary impedance, *f* is the frequency, *C* is the capacitance and *C*_sp_ is the specific capacitance*.* The capacitance and specific capacitance at 0.5 V calculated from the *Z*′′ values at the high frequency (*f* = 100 kHz) were found to be 85.2, 92.76 and 169–222 F g^−1^, respectively. The results are in agreement with fitted values, CV and GCD. Conclusively, the prepared electrochemical capacitive transducer (Ni/Cu/Cu_2_O/CuO/ZnO) possesses potential energy storage and other energy applications.

## Conclusions

To the best of our knowledge, a first-time facile strategy to prepare Cu/Cu_2_O, ternary Cu/Cu_2_O/CuO/ZnO nanocomposite and Cu/Cu_2_O/CuO/ZnO structured electrode was developed by the green route, which avoided tedious synthetic methods. The SEM images revealed that the temperature played a pivotal role in different morphological structure of the nanomaterials. The results prove that the green route is the ease of use and can be successfully employed in the preparation of different metal oxides. In the interim, TEM and XRD techniques were able to determine the crystallinity and particle size of the as-synthesised ternary nanocomposite while *Punica granatum* L peel extract used as a capping agent render the nano-oxides soluble and stable. The retention and mechanism of interaction of the capping ligand on the metal precursor surface was successfully confirmed by FTIR and UV-Vis studies, evident by change in vibrational stretches related to C–H, C=O and –O–H functional groups and formation of the reduced Cu/Cu_2_O/CuO/ZnO nanocomposite. The electrochemical and catalytic activity of the prepared nanocomposite was determined by EIS, CV and GCD. The retentivity of the capacitance at the electrode interface after several cycles was found to be stable. The Ni/Cu/Cu_2_O/CuO/ZnO electrode reached a significant maximum *C*_s_ of 241 F g^−1^, *E*_d_ of 32 Whkg^−1^ and *P*_d_ of 65 Wkg^−1^. The Cu/Cu_2_O/CuO/ZnO structured electrode exhibits large surface area and shows a high electrocatalytic behaviour and accessibility for ion diffusion. Further, UV-Vis and EIS provided the conductivity of the nanocomposite which was presented by smaller *R*_ct_, *f*_low_ and smaller *E*_g_. In this view, we found the Ni/Cu/Cu_2_O/CuO/ZnO system to be more superior compared to the mono-metallic oxides (CuO, ZnO, etc.), binary oxides (CuO/ZnO) and some ternary oxides. The results advocate efficient employment of nanoplatelets structured electrode in the fabrication of supercapacitor devices. Further, the green prepared nanocomposite opens a wide variety of applications due to their structural, catalytic and capacitive nature. Thus, if modified, the semiconducting nanoplatelets structured electrode will be a potential candidate in various fields of application such as photocatalysis, antibacterial activity and energy storage devices.

## Abbreviations

*C*: Faradaic pseudo-capacitance
*C*_s_: Specific capacitance
CV: Cyclic voltammetry
EIS: Electrochemical impedance
FTIR: Fourier transform infrared spectroscopy
GCD: Galvanostatic charge-discharge
HRSEM: High-resolution scanning electron microscopy
HRTEM: High-resolution transmission electron microscopy
PL: Photoluminescence
*R*_ct_: Charge-transfer resistance
Rs: Solution resistance
*W*: Warburg diffusion impedance
XRD: X-ray diffraction
